# Fretting Wear Damage Mechanism of Uranium under Various Atmosphere and Vacuum Conditions

**DOI:** 10.3390/ma11040607

**Published:** 2018-04-16

**Authors:** Zhengyang Li, Zhenbing Cai, Yanping Wu, Xiandong Meng, Dongxu Zhang

**Affiliations:** 1 Tribology Research Institute, Key Lab of Advanced Technologies of Materials, Southwest Jiaotong University, Chengdu 610031, China; lzy_jiaoda@126.com(Z.L.); 2 China Academy of Engineering and Physics, Mianyang 621900, China; lsb371698207@163.com (X.M.); wusongbow6@foxmail.com (D.Z.)

**Keywords:** fretting wear, wear mechanism, atmosphere, uranium

## Abstract

A fretting wear experiment with uranium has been performed on a linear reciprocating tribometer with ball-on-disk contact. This study focused on the fretting behavior of the uranium under different atmospheres (Ar, Air (21% O_2_ + 78% N_2_), and O_2_) and vacuum conditions (1.05 and 1 × 10^−4^ Pa). Evolution of friction was assessed by coefficient of friction (COF) and friction-dissipated energy. The oxide of the wear surface was evaluated by Raman spectroscopy. The result shows that fretting wear behavior presents strong atmosphere and vacuum condition dependence. With increasing oxygen content, the COF decreases due to abrasive wear and formation of oxide film. The COF in the oxygen condition is at least 0.335, and it has a maximum wear volume of about 1.48 × 10^7^ μm^3^. However, the COF in a high vacuum condition is maximum about 1.104, and the wear volume is 1.64 × 10^6^ μm^3^. The COF in the low vacuum condition is very different: it firstly increased and then decreased rapidly to a steady value. It is caused by slight abrasive wear and the formation of tribofilm after thousands of cycles.

## 1. Introduction

Uranium has been extensively applied in nuclear power plants and nuclear devices due to its special material and nuclear properties [1]. To satisfy the engineering design, uranium will inevitably contact or fasten with other materials, among which the contact interface has been proved to bear different stresses and relative clearances. While in transport and service, uranium is frequently affected by external excitation such as vibration, thermal cycling, and dry-wet alternation, which could lead to relative motion with small displacement amplitude, and the relative motion will cause fretting damage in the contact interface, thus accelerating fatigue crack initiation and propagation [2,3,4]. Considering that fretting damage can significantly decrease fatigue life [5], the fretting wear of contact interface plays a vitally importance role in the mechanical behavior and needs to be investigated thoroughly.

Based on the direction of relative motion, four basic fretting modes can be summarized, which are tangential fretting, radial fretting, rotational fretting, and torsional fretting [6,7]. To properly describe fretting behaviors, the concept of fretting map (tangential force plotted versus displacement amplitude) was first proposed by Vingsbo et al. [8], namely stick regime, mixed stick-slip regime, and gross slip regime. Fretting theory was further developed into two kinds of fretting maps by Zhou et al. [9] and Vincent et al. [10], that is, running condition fretting map (RCFM) and materials response fretting map (MRFM) respectively. The fretting map was based on experimental studies conducted on an aluminum-lithium alloy under different displacement, load, and frequency.

Researchers have investigated fretting damage and its characterization under air, solution, and high temperature conditions [11,12,13,14,15]. Limited work has been carried out to characterize fretting damage under vacuum or other atmosphere conditions. However, material and atmosphere conditions play an important role in failure mechanism due to fretting [16,17]. Chaudhry [18,19] investigated the fretting damage mechanism of stainless steel and chromium carbide coating in vacuum and air conditions. Results show that damage mechanisms involved in vacuum is entirely different to those observed under air conditions. Uranium is usually served in a nuclear power plant and nuclear devices conditions [20]. The low oxygen content and vacuum condition is similar to a fast breeder reactor and fusion reactor condition. Considering that the atmosphere conditions play an important role in the failure mechanism due to fretting, it is necessary to identify the different damage mechanisms under different atmosphere and vacuum conditions. Studying the fretting wear behavior of uranium under different atmosphere and vacuum conditions will help to provide some guidance for design and application of uranium.

These facts led the authors to focus on the current study, which involves the fretting wear behavior of uranium under different atmosphere and vacuum conditions. Attention will be given to the coefficient of friction (COF), morphology, oxide, and microstructure of wear scar, which is used to characterize the fretting damage mechanism.

## 2. Experimental Procedure

### Materials and Specimens

**Figure 1:**
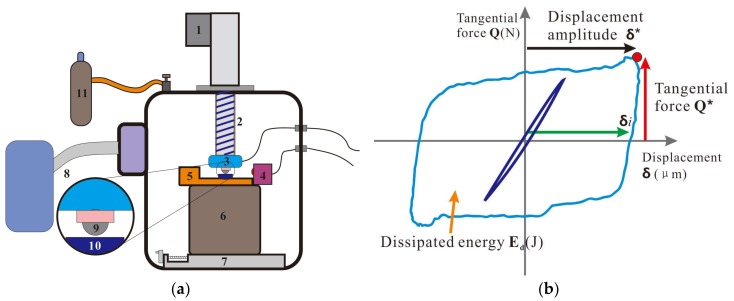
Schematic view of experimental equipment (**a**) and fretting loop analysis (**b**). (**1**) servo electric cylinder; (**2**) lead screw; (**3**) three-dimensional pressure sensor; (**4**) laser copolymerization sensor; (**5**) piezoelectric ceramics; (**6**) base; (**7**) screw module; (**8**) vacuum pump system; (**9**) ball sample; (**10**) plate sample; (**11**) gas transmission system.

The fretting wear test was carried out on a ball-on-disk configuration. The upper ball sample was AISI52100 steel with a diameter of 12 mm, a hardness of 766 HV, and a roughness of Ra = 0.2 μm, Rz = 0.6 μm, Rpk = 0.3 μm. The samples were purchased from Jinfu Mechanical and Electrical Center (Chengdu, China). Because the bearing steel is always used as a mechanical part to bear stress and contact with uranium in depleted uranium armor and nuclear weapon, we used the bearing steel as a tribological pair. The lower plate sample was uranium with a roughness of Ra = 1.5 μm, Rz = 6.1 μm, Rpk = 1.8 μm. A schematic drawing of the fretting wear test setup and sample is shown in Figure 1a.

The normal force P was applied directly on the upper sample holder using a servo electric cylinder. The normal force P and tangential force Q was measured by three-dimensional pressure sensor. The relative displacement δg was imposed by a piezoelectric ceramic and measured by a laser copolymerization sensor with an accuracy of ±1 μm. A base was designed to support the test apparatus and adjust the position of lower sample. The atmosphere condition was controlled by a pump system and gas transmission system. During the test, the normal force P, tangential force Q, COF, displacement amplitude δg as well as number of cycles, were recorded and compared at the same time. These variables were used to draw fretting loops for a constant normal force condition (Figure 1b). The diagram of a gross sliding fretting loop with the key parameters indicated: Q*, tangential force; δ*, sliding amplitude; δ*i*, interfacial displacement amplitude; and Ed (J), dissipated energy.

The test parameter is listed in Table 1. The displacement amplitude of 100 μm together with a frequency of 10 Hz was chosen for the tests. The normal load was 20 N, realizing a mean contact pressure of about 700 MPa evaluated by Hertzian contact theory. The duration was set to 10,000 cycles. Different atmosphere (Ar, Air (21% O_2_ + 78% N_2_), and O_2_), low vacuum (LV), and high vacuum (HV) conditions were investigated in this study. Each test was repeated three times.

After the fretting tests, the wear scar of uranium was directly analyzed by Raman spectrum (Lab Ram HR, Horiba Ltd., Kyoto, Japan) characterization. The analyzed area included the center and edge of the wear scar. After this, the samples were cleaned with acetone and ethanol, and then dried. The morphology of the wear scar was analyzed by scanning electron microscopy (SEM, FE SEM 20, JEOL, Tokyo, Japan). The focused ion beam (FIB, JEOL, Tokyo, Japan) was used to analyze the damage of cross section. The 3D surface measurement instrument (Contour GT X3, Bruker, Karlsruhe, Germany) is used to better understand surface damage after removing the majority of adherent wear debris, which includes 2D profile and wear volume of wear scar. The wear volume was directly calculated by software using a 3D surface measurement instrument. The wear volume is below the 0-lever or the substrate surface.

## 3. Results and Discussion

**Figure 2:**
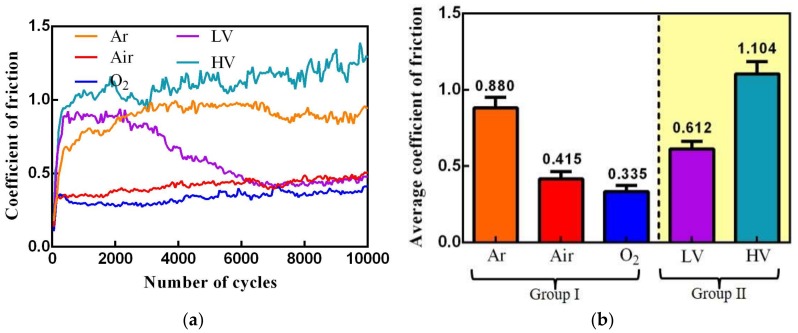
The COF versus cycle number (**a**) and the average COF under different environment conditions (**b**).

The various COFs for uranium under different atmosphere and vacuum conditions are presented in Figure 2a. The average COF is shown in Figure 2b. It can be observed that a COF with a number of cycles goes through dramatic changes in the early stage, which presents a running-in process. At the start of the running-in process, the polished interfaces of uranium specimens begin to contact and slide with the friction counterparts. The damage on the interfaces resulting from fretting in the running-in process are not obvious, so the COF firstly presents very low values due to the smoothness of polished interfaces. After this stage, the reciprocating friction increases the surface roughness, which leads to the COF increase. The COF will not attain a steady value until the interfaces of uranium specimens and friction counterparts reach a dynamic balance. With increasing oxygen content, the COF decreases. With increasing vacuum, the COF increases. Because the existence of oxygen would contribute to forming tribofilm or oxide film in interface to reduce friction, a detailed analysis would be summarized later. When the test condition is HV, average COF value is more than 1, which is caused by severe adhesive wear and seizure of the contacting material [18]. The COF change in the LV condition is very different, which firstly increases and then decreases rapidly to a value similar to that obtained in Air condition. The high COF in the initial stage is caused by adhesive wear, because the contact interface has little oxygen and water. As the fretting process goes on, the temperature in friction interface increases due to the friction heat [21]. The friction heat will contribute to the tribo-chemical reaction of uranium with little oxygen or water. The tribo-chemical reaction will lead that the contact interface generating oxide debris, and the oxide debris will change the wear mechanism from adhesive wear to abrasive wear. Meanwhile the existence of oxide debris avoids the direct contact of the friction pair. As a result, the COF decreases. These results are in accordance with the previous study reported by another group [22,23].

**Figure 3:**
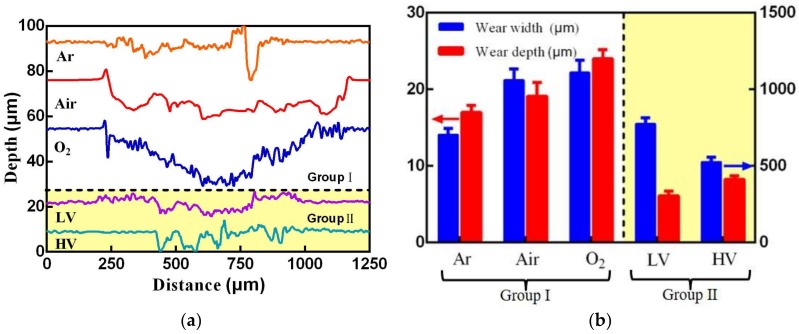
The profile micrographs of wear scars (**a**) and the corresponding wear width and wear depth (**b**) under different atmosphere and vacuum conditions.

Figure 3a shows the scar profile of uranium surface under different atmosphere and vacuum conditions. The scar profile is parallel to the fretting direction. The profile under Air, O_2_, and LV condition shows a “U” shape. However, a “W” shape is observed under Ar and HV condition, which is caused by severe adhesive wear. The upper sample’s material unidirectionally transfers to the lower sample and leads to a bulge in the wear scar center. Figure 3b shows the wear width and wear depth under different atmosphere and vacuum conditions. With increasing oxygen content, the wear width and depth increase. A minimum wear depth of about 6.1 μm occurs under the LV condition, which is far lower than that of Air condition (24.1 μm). Under the O_2_ condition, the wear scar shows a maximum wear width of about 1110.5 μm.

**Figure 4:**
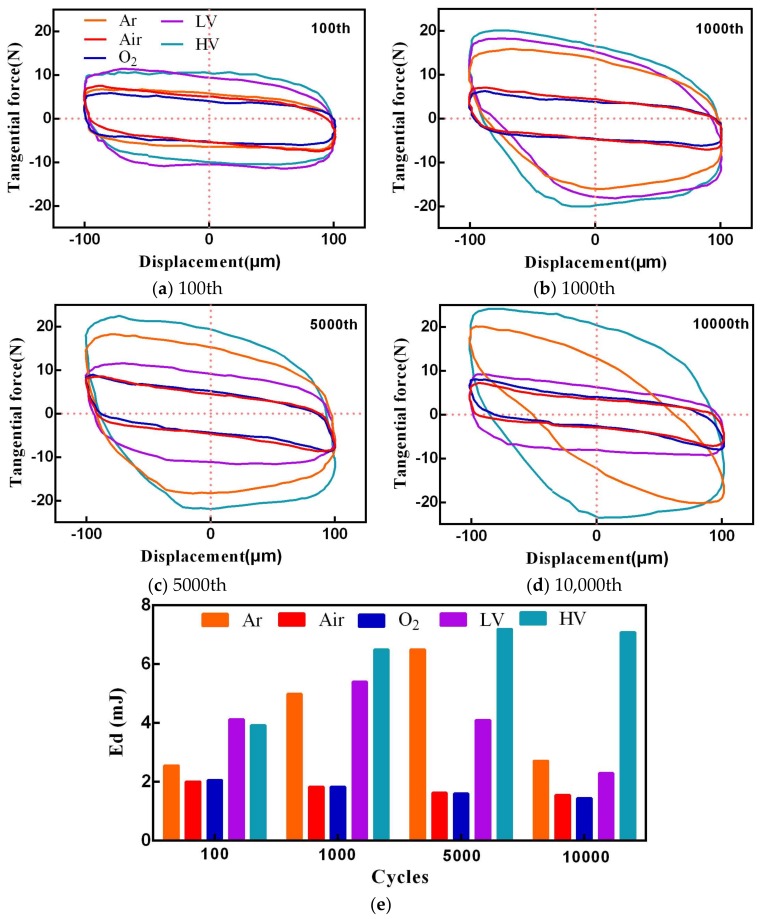
Evolution of fretting loops with cycle number under different atmosphere and vacuum conditions (**a**–**d**) and Ed of each cycles (**e**).

Running condition fretting loops are the most essential pieces of kinetic information. Figure 4a–d shows a typical representation of fretting loops as a function of cycle number from 1 × 10^2^th to 1 × 10^4^th under different atmosphere and vacuum conditions. In all cases, the loop shape remains approximately constant during the entire test, and presents a parallelogram shape, which implies that fretting is running in the gross slip condition and the slip extends to the entire contact area. A good symmetry was observed for the fretting loops. The area of fretting loop indicates the friction dissipated energy (Ed). Figure 4e shows Ed of each cycles. However, the fretting loops under the Ar and HV conditions exhibit a very high tangential force and Ed. Existence of this phenomenon indicates that crack-like damage and adhesion would occur at the contact interface due to severe shearing under cyclic loading, and finally propagates under fatigue action [19]. Adhesion at the contact interface indicates the occurrence of material transfer between the friction pair. In addition, Ed (Air and O_2_) has almost no change with the increase of cycle number. In each cycle, the Ed under the HV condition shows the biggest value, and the Ed in O_2_ condition is lowest.

**Figure 5:**
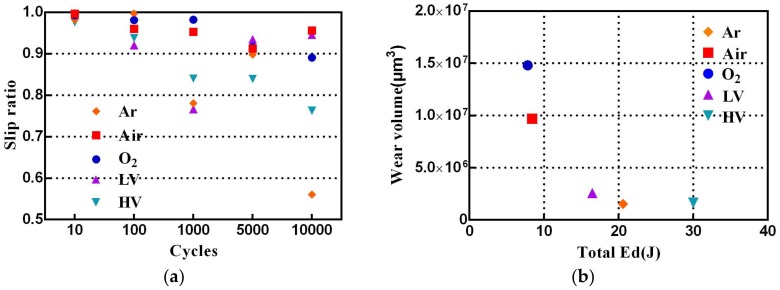
Evolution of slip ratio with cycle number (**a**) and Ed versus wear volume under different atmosphere and vacuum conditions (**b**).

More differences between the fretting loops under a different atmosphere and vacuum conditions can be found based on the slip ratio and total Ed. The slip ratio is defined as the ratio of δ*i* and δ* [24]. Figure 5a shows variation of the slip ratio under different atmosphere and vacuum conditions. It can be found that the slip ratio in Air and O_2_ condition always exhibits a very high value. Higher slip ratio indicates existence of more interfacial slip at contact interface [18]. This variation has been attributed to metal flow at the contact interface. Figure 5b shows the total Ed versus wear volume under different atmosphere and vacuum conditions. The total Ed over the test is the sum of Ed for the individual fretting cycles. As can be seen in Figure 5b, the total Ed is arranged in order as follows: O_2_ (7.80 J) < Air (8.42 J) < LV (16.48 J) < Ar (20.58 J) < HV (30.02 J). The low oxygen condition (Ar, LV, and HV) shows a very low wear volume, and the high oxygen condition exhibits a very high wear volume due to elevated oxidation and abrasive wear.

**Figure 6:**
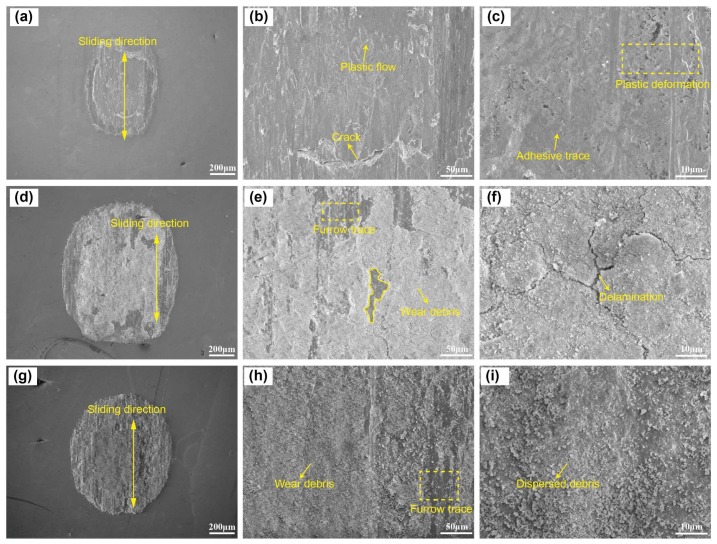
The SEM morphology of worn surface under Ar (**a**–**c**), Air (**d**–**f**), and O_2_ (**g**–**i**) conditions.

Wear scars of uranium specimens are investigated by SEM to obtain further revealing of the tribological mechanisms. The wear scar of uranium specimens under Ar, Air, and O_2_ conditions are shown in Figure 6a–i, respectively. Compared to Air and O_2_ condition, the uranium surface is polished by the relative sliding against friction pair, resulting in a smooth wear scar, whereas no wear debris is distributed under the Ar condition (Figure 6a). A large crack of more than 100 μm is clearly observed in Figure 6b, and wear scar appears to exhibit plastic flow under cyclic shear stress. Plastic deformation and adhesive trace can be observed in Figure 6c. Typical characterization of adhesive wear is slight damage and high COF. Hence, the COF under Ar condition is very high. The extreme adhesion tendency induces that the upper ball sample (or debris particles) adheres to the uranium surface, which also increases the plastic deformation in the contact and eventually leads to reduced interfacial wear [25,26]. Hence, the low wear volume is obtained under the Ar condition (Figure 5b).

In the Air condition, the wear surface is covered by much lamellar wear debris and an evident furrow trace with different depths and widths is observed in Figure 6d. Part of the wear debris is compacted into a discontinuous oxide film, while the other part is stripped out from the wear surface and a matrix emerges, presenting an apparent scar contour (Figure 6e) [27,28,29]. On the other hand, the scar contour could be caused by the delamination of the oxide film under shear stress. The delamination trace of the oxide film can be observed in Figure 6f, suggesting that abrasive wear is the primary mechanism of wear in this condition, or at least the most significant one.

In the O_2_ condition, the oxide film presents a shape of rough surface throughout most of the wear surface (Figure 6g). Many sections of small and uniform wear debris form and adhere to the wear scar. Plowing partly appears at the oxide film, which is a typical characteristic of abrasive wear (Figure 6h). The wear debris is fully grinded during fretting, and this type of wear debris detaches from the surface typically with a few microns (Figure 6i). However, the oxide film under the O_2_ condition is not compacted compared to the air condition due to the absence of water, even though the oxide film is compacted, the impacted debris is broken up again because of weak adhesion [30]. Oxidation wear and abrasive wear is the main wear mechanism in the O_2_ condition. Hence, with increasing oxygen content, the wear mechanism changes from adhesive wear to abrasive wear and oxidation wear. The oxide film occurs at the friction interface, which directly avoids contact of the two materials. The COF decreases and wear volume increases with increasing oxygen content.

**Figure 7:**
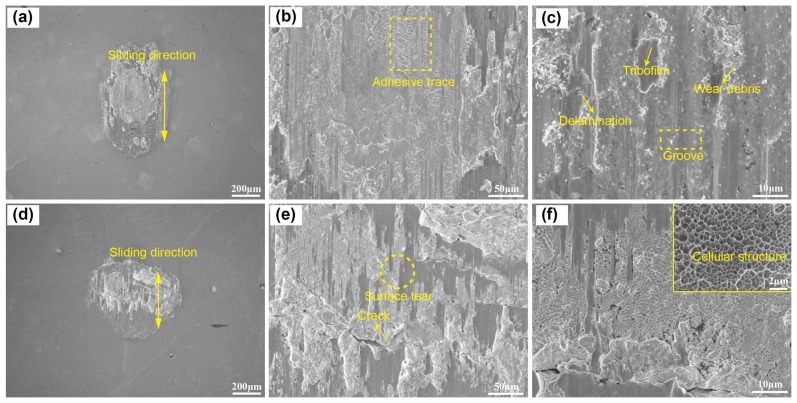
The SEM morphology of worn surface under LV (**a**–**c**) and HV (**d**–**f**) conditions.

The wear scar of uranium specimens under LV and HV conditions are shown in Figure 7. In the LV condition, it is evident that material is transferred from ball to flat or conversely in the center of the contact interface, and the edge of wear scar shows a scratching surface in Figure 7a. Characteristic wear debris, matrix torn up, groove, adhesive trace, and micro-cracks occur at the wear surfaces (Figure 7b). The wear particle distributes around the tribofilms and leads to slight third-body abrasive wear, which also cause plowing grooves, as shown in Figure 7c. The existence of tribofilms would reduce COF. Hence, the COF decreases after 2000 cycles in Figure 2a. The wear mechanism under the LV condition is severe adhesive wear and slight abrasive wear [22,23].

A similar morphology with severe surface damage was observed in the form of plastic deformation, fracturing of the surface, and material transfer under the HV condition in Figure 7d. Many discontinued cracks were observed in the wear scar in Figure 7e, presenting a large area of cavities. The contact center forms slip bands due to intense shearing under cyclic loading, and the existence of fatigue striations on the surface of cracks indicates the possibility of crack initiation due to severe plastic deformation and crack propagation under fatigue action [18]. Part of the wear scar shows a smooth surface, while another part presents a rough surface. A higher-magnification micrograph reveals the damage mechanism (Figure 7f) at the rough damaged surface, which shows a cellular structure due to local adherence in the friction interface tearing the substrate surface by shear stress, just like the fracture morphology [31]. Under the vacuum condition, the absence of oxygen gives rise to adhesive wear such that the wear volume is quite small, because the material is adhered to the surface and remains within the contact [22]. Hence, with increasing vacuum degree, the wear mechanism changes from severe adhesive wear and slight abrasive wear to fatigue wear. Slight abrasive wear and part tribofilm in the LV condition reduces the friction. The COF increases and the wear volume decreases with increasing vacuum degree.

**Figure 8:**
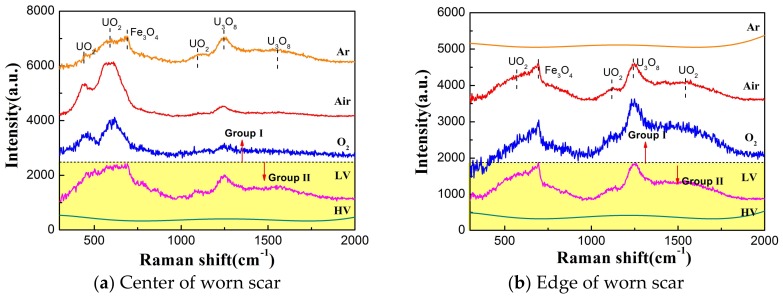
The Raman spectrum of the wear surface under different atmosphere and vacuum conditions.

**Figure 9:**
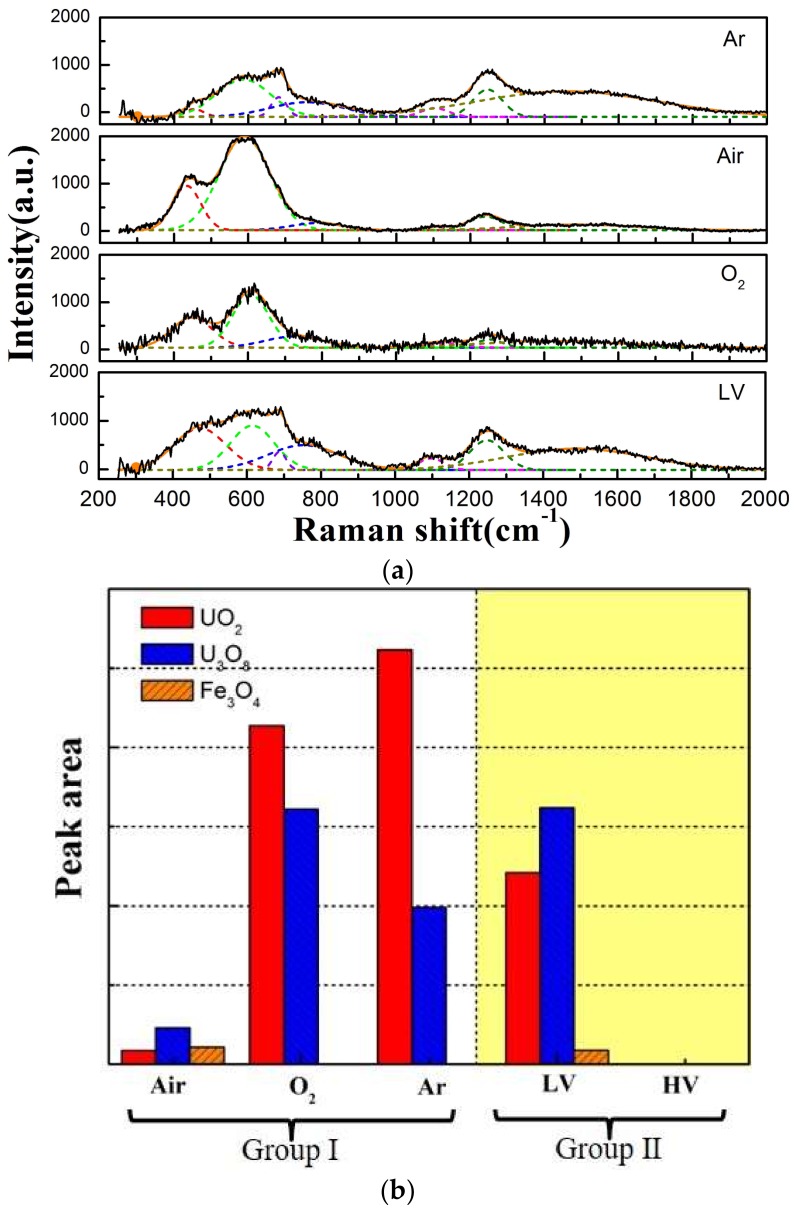
Representative Raman spectra with spectral peak fitting for center of worn surface (**a**) and the peak area (**b**).

As described above, the wear surface is mainly composed of many sections of oxide wear debris and transfer material. Figure 8a,b shows the Raman spectrum of wear surface under different atmosphere and vacuum conditions to determine the chemical composition. The Raman peaks at 445, 576, and 1150 cm^−1^ agree with the Raman shifts of UO_2_, which is the stretching vibration of U-O. The Raman peaks at 752, 1250, and 1522 cm^−1^ are associated with U_3_O_8_, as has been reported [32,33]. In the center of the wear surface, it can be seen from Figure 8a that the feature Raman peaks of Fe_3_O_4_ (675 cm^−1^) appear after sliding against the ball in the Ar and LV condition, which indicates that material transfer exists in the fretting process [34]. We guess that the partial oxidation of Fe_3_O_4_ and U_3_O_8_ particles come up after the test, because the chemistry of uranium is very active, and it can react with oxygen in a very short time [35]. Under the HV condition, no obvious Raman peaks appear in the center of the worn surface, which maybe formed amorphous oxide and could not be detected by the Raman spectrum. However, the dominant Fe_3_O_4_ and U_3_O_8_ oxide cover the edge of wear surface. To compare the Raman spectra of different samples, Gaussian fitting is applied to represent the most probable chemical component in Figure 9.

**Figure 10:**
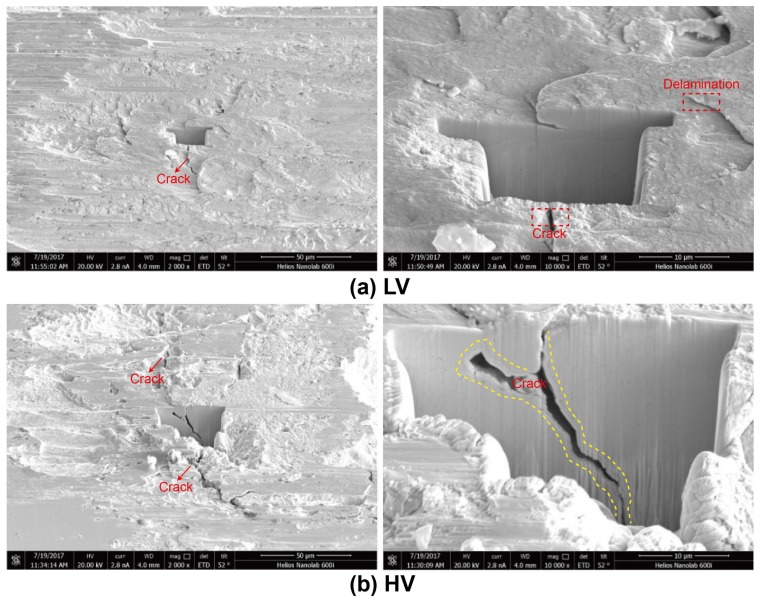
Cross-section images of the worn surface under different vacuum conditions. (**a**) LV; (**b**) HV.

To check the damage mechanism of interface under the vacuum condition, an ion beam milling operation has been carried out using FIB, as shown in Figure 10. A clear demarcation line at the interface can be seen. From Figure 10a, severe plastic deformation and micro-cracks are observed, and the crack forms beneath the contact interface due to the maximum shear stress occurring at the sub-surface region [36]. A very deep crack of more than 40 μm and rough wear surface is observed in Figure 10b. It is observed that the nucleation of the crack would be governed by severe shear stress, and the propagating degree of the crack would increase with the increase of the vacuum degree. Another important point to note is that the crack would finally lead to the separation of the adhesive bodies beneath the contact interface.

**Figure 11:**
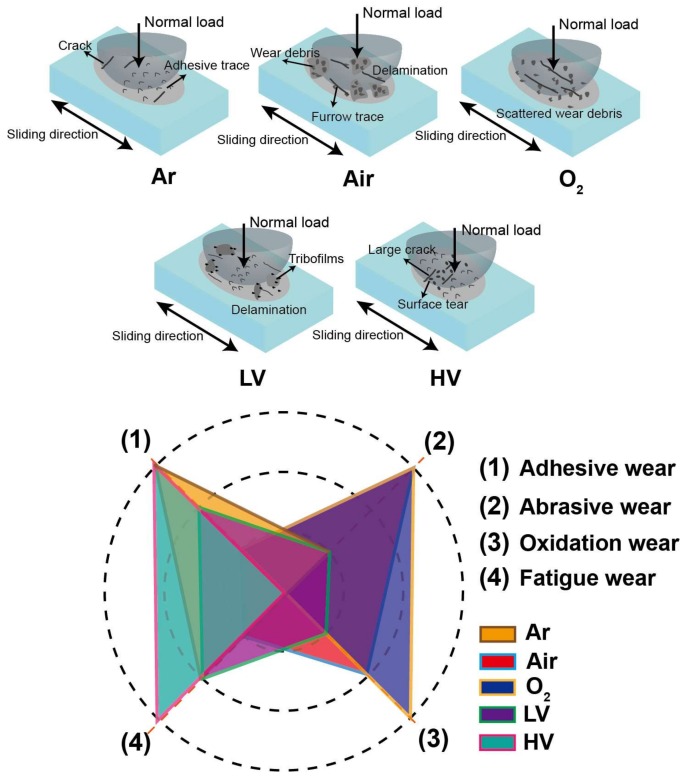
Schematic diagrams of the wear model and wear mechanism of uranium under different atmosphere and vacuum conditions.

The wear mechanism under different atmosphere and vacuum conditions can be described by schematic diagrams, as shown in Figure 11. Four wear mechanisms—abrasive wear, adhesive wear, oxide wear, and fatigue wear—are used as evaluation indexes and roughly divided into three grades—I, II, and III—where I is inferior and III is superior. In the Air and O_2_ conditions, oxidation wear and abrasive wear is the main wear mechanism due to wear debris and furrow trace. In Ar and HV conditions, the adhesive trace and cracks show the adhesive wear and fatigue wear is the main wear mechanism. Meanwhile, in the LV condition, the formation of tribofilms reduces the COF, and the wear mechanism induces adhesive wear, fatigue wear, and slight abrasive wear.

## 4. Conclusions

Fretting wear behavior of uranium under different atmosphere and vacuum conditions was investigated. The wear mechanism of uranium under different atmosphere and vacuum conditions were discussed. The main conclusions are as follows:

(a) The COF in oxygen condition is at least about 0.335, and it has a maximum wear volume of about 1.48 × 10^7^ μm^3^. However, the COF in high vacuum condition is at most about 1.104, and the wear volume is 1.64 × 10^6^ μm^3^.
(b) The COF in a low vacuum condition is very different, which firstly increases and then decreases rapidly to a steady value. This is caused by partly abrasive wear and the formation of tribofilm after thousands of cycles.
(c) With increasing vacuum degree, the COF increases and the characteristic of plastic deformation, crack, and adhesive trace becomes more obvious. With increasing oxygen content, the COF decreases due to abrasive wear and the formation of oxide film. However, the wear volume increases with increasing oxygen content.

## Figures and Tables

**Table 1:** Parameter of the fretting wear test.

Group	I			II	
Specimen	1#	2#	3#	4#	5#
Atmosphere	Ar	Air (21% O_2_ + 78%N_2_)	O_2_	Air	
Pressure (Pa)	1.01 × 10^5^			1.05	1 × 10^−4^
Displacement amplitude (μm)	100			100	
Load (N)	20			20	
Cycle	10^4^			10^4^	
